# Navigating barriers and building solutions: a mixed-methods study on sexual and reproductive healthcare for migrant women in Milan

**DOI:** 10.1017/S1463423626100954

**Published:** 2026-02-27

**Authors:** Giacomo Marro, Eleonora Splendi, Giulia Russo, Anastasia Scher, Emanuele Longo, Davide Giacomino, Loredana Carpentieri, Alessia Mancuso-Prizzitano, Monica Trentin, Alessandro Lamberti-Castronuovo

**Affiliations:** 1 EMERGENCY ONG ETS, Italy; 2 CRIMEDIM - Center for Research and Training in Global Health, Humanitarian Aid, and Disaster Medicine - Università degli Studi del Piemonte Orientale Amedeo Avogadro, Novara, Italy

**Keywords:** Barriers to access, EMERGENCY NGO, Italy, migrant women, sexual and reproductive health

## Abstract

**Aim:**

To develop strategies to lower barriers to sexual and reproductive health (SRH) care for migrant women (MW) in Milan, Lombardy, Italy.

**Background:**

SRH is a fundamental human right, yet MW experience poorer SRH outcomes than non-MW due to cultural, linguistic, legal, and financial barriers. Despite Italy’s universal healthcare system (*Servizio Sanitario Nazionale*, SSN), disparities persist.

**Methods:**

Quantitative SRH data from the health information system of a non-governmental organization (NGO) clinic in Milan was used to describe the demographic, socioeconomic, and administrative profile of MW with SRH needs, and to examine factors associated with SRH-related consultations. Qualitative data were collected through semi-structured interviews with 29 stakeholders, including MW, healthcare workers, NGO representatives, and policymakers. Thematic analysis was guided by a socio-ecological framework across individual, organizational, societal, and policy levels.

**Findings:**

SRH needs were the most frequent presentations among MW accessing the clinic. Most MWs came from Romania, Morocco, and Peru. Nearly half of those eligible for SSN registration were not enrolled, primarily due to lack of awareness. Economic vulnerability was strongly linked to SRH needs, while language proficiency alone showed no significant effect. Interviews underscored the importance of culturally sensitive care and mental health support. They also emphasized the inconsistent enforcement of regulations across government facilities and legislative gaps that leave certain groups, particularly undocumented EU nationals, without essential services. Community networks and stronger coordination across providers - including formal collaboration between NGOs and the SSN - were identified as promising levers to improve SRH access and equity in Milan and similar settings.

## Introduction

Sexual and reproductive health (SRH) is a global health priority and a fundamental human right (World Health Organization, 2012). Yet this right is unequally upheld for vulnerable groups (UNFPA, 2014; UNFPA, 2019), including migrant women (MW), who face poorer SRH outcomes and limited access to services due to linguistic, cultural, financial, and legal barriers (Keygnaert *et al.*, 2014; Mandroiu *et al.*, 2024). Undocumented women are particularly affected, experiencing higher rates of induced abortions, caesarean sections, and complications, alongside lower utilization of services and limited awareness of their rights (Crenshaw, 1991; Jacquemyn *et al.*, 2012; Pérez-Sánchez *et al.*, 2024).

The WHO European region hosts over 100 million migrants, including over 12 million refugees (World Health Organization, 2023), half of whom are women and girls (McAuliffe and Oucho, 2024). In Italy, migrants represent 8.7% of the total population, with Lombardy as the main host region (ISTAT, 2023; Caritas Italiana and Fondazione Migrantes, 2023; ORIM, 2021). Milan alone accounts for 40.6% of Lombardy’s foreign residents, including approximately 44,000 undocumented individuals, nearly half of whom are women (CGIL and UIL, 2021; ORIM, 2021).

Italy’s National Health Service (*Sistema Sanitario Nazionale*, SSN) is based on universal coverage and equal access, as guaranteed by the Italian Constitution (Costituzione della Repubblica Italiana, 2023). Yet, migrants often face health disparities based on their legal status. While asylum seekers and refugees have the same rights as Italian citizens (Testo Unico Immigrazione – TUI. Consolidated Act on Immigration, 1998), around 30% of residence permit holders are not registered (OSAR, 2021). Undocumented migrants are entitled to urgent and essential care (Decreto legislativo, 2013; Circolare n. 4, 2008), including most SRH services, but unclear definitions and regional disparities limit access. In Lombardy, for example, undocumented EU nationals are excluded from ENI code^1^ coverage, leaving them without effective access to care (Circolare n. 4, 2008). These legal and administrative complexities are only one dimension of a broader set of social determinants of health (SDH), such as housing instability, precarious work, and linguistic exclusion, that further exacerbate barriers for MW (World Health Organization, 2008; Egli-Gany *et al.*, 2021).

Access to SRH services may be challenging for all women in Italy (Human Rights Watch, 2021), but it is particularly difficult for MW. Difficulties are reported in abortion care, contraception, and cancer screening (Damiani *et al.*, 2012, Lariccia *et al.*, 2013; Pérez-Sánchez *et al.*, 2024), as well as in maternal health, where MW often experience delayed prenatal visits, fewer recommended tests, and poorer neonatal outcomes (Lauria *et al.*, 2013; Chiavarini *et al.*, 2016; Di Napoli, 2022). These challenges are compounded by administrative and cultural barriers that complicate navigation of the SSN (Damiani *et al.*, 2012; Lauria *et al.*, 2013; Lauria *et al.*, 2013; Chiavarini *et al.*, 2016; Di Napoli, 2022). With limited access to regular public health services, a significant portion of care for this population is delivered by volunteer-based or non-governmental organizations (NGOs) which provide valued services but often operate parallel to the SSN, limiting continuity of care and prioritizing acute needs over integrated, long-term healthcare solutions (World Health Organization, 2018; Mandroiu *et al.*, 2024).

Despite a growing body of European research on migrant health (Sami *et al.*, 2019; López-Domene *et al.*, 2019; Chiarenza *et al.*, 2019; Funge *et al.*, 2020; Bains *et al.*, 2021; Nellums *et al.*, 2021), studies focusing specifically on SRH access among MW in Italy remain limited. Existing literature addresses individual or structural barriers (Meier *et al.*, 2022; Mazzitelli *et al.*, 2025), or specific clinical aspects (Fontanelli Sulekova *et al.*, 2021; Rubini *et al.*, 2024). However, few studies adopt an interdisciplinary perspective that integrates clinical data with social and policy analysis, and mixed-methods approaches providing both clinical data and stakeholder perspectives remain scarce (Human Rights Watch 2021, Mandroiu *et al.*, 2024).

This study addresses these gaps by providing granular health data from an NGO-run primary care clinic in Milan, Italy, and by contextualizing the findings with insights into the barriers to SRH care, informed by perspectives from MW, along with insights from relevant local stakeholders. By combining these perspectives, the study develops evidence-based recommendations to improve SRH access and equity for MW in Milan, with potential relevance for other urban contexts.

## Methodology

This study employs a mixed-methods approach, combining quantitative analysis of an electronic dataset from an NGO-run primary care clinic with qualitative insights from semi-structured interviews. Using a concurrent design (Cresswell, 1999), this approach facilitates triangulation, enriching the understanding of barriers to access to SRH care in Milan.

### Quantitative study

#### Data source and study population

The quantitative analysis in this study is based on anonymized data from EMERGENCY, an international humanitarian NGO operating in Milan through a mobile clinic and an outpatient programme (Emergency NGO ETS, 2025). These services offer patients bureaucratic, legal, and cultural support to facilitate access to the SSN. When patients are unable to access care through the SSN, basic primary care is provided directly. All patient encounters are recorded electronically, capturing sociodemographic, administrative, and clinical information, as well as the specific types of support received (e.g., administrative assistance, medical consultation). Multiple services can be provided during a single visit.

The dataset includes all female patients who accessed EMERGENCY’s services in Milan between July 1, 2017, and December 21, 2023. Until now, gender has been recorded based on self-report, with only male and female options available in the system. This analysis focuses on MW, defined here as women with a migratory background, including both those born abroad and those born in Italy to migrant parents, who sought care for SRH-related diagnoses (i.e., SRH group). This categorization reflects the layered and generational dimensions of migration and acknowledges the unique experiences and barriers these individuals may face in accessing SRH care, in line with the study’s focus (Rogers *et al.*, 2023). SRH diagnoses were identified using the International Classification of Diseases (ICD-10) (International Classification of Diseases, n.d.) and criteria detailed in Table 1. Only first-time SRH-related diagnoses were included. Diagnoses were recorded during medical consultations, with up to three documented per visit. For follow-up visits, a generic ICD code replaced the initial condition-specific one.

**Table 1.** Study population (SRH group): inclusion and exclusion criteria of the quantitative analysis

	Inclusion criteria	Exclusion criteria
**Population**	Female gender	Male gender or gender missing/not declared
**Timeframe and geographic location of access to NGO services**	Access to NGO services between 1st July 2017 and 21st December 2023 in Milan, Italy	Access occurred before 1st July 2017 or after 21st December 2023 and/or at a different NGO service location
**Type of service accessed**	Medical evaluation	Other services (nursing care, cultural mediation, administrative support…)
Sexual and Reproductive Health (SRH) needs*	At least one of the following diagnoses assigned at the end of medical evaluation: - Mild mental and behavioural disorders associated with the puerperium, not elsewhere classified (code F53, chapter V) - Habitual aborter (code N96), or Female infertility (code N97) or Complications associated with artificial fertilization (code N98) [chapter XIV] - Any of the diagnoses included in chapter XV (Pregnancy, childbirth and the puerperium) - Termination of pregnancy, affecting foetus and newborn (code P96.4, chapter 16) - Diagnoses assigned in case of ‘Persons encountering health services in circumstances related to reproduction’ (codes Z30-Z39, chapter XXI) - Problems related to unwanted pregnancy (code Z64.0) or Problems related to multiparity (code Z64.1) [chapter XXI]	Other gynaecological diagnoses, such as: - Benign and malignant neoplasms of female genital tract (codes D25-D28 and C51-C58) - Inflammatory diseases of female pelvic organs (codes N70-N77) - Most noninflammatory disorders of female genital tract, including menstrual cycle alterations (codes N80-N95)
**Timing of SRH need evaluation**	First medical evaluation for at least one SRH-related need	Follow-up medical evaluation for SRH-related needs, with no new SRH-related condition diagnosed.

* *SRH need is defined according to the diagnoses associated with the medical evaluation and coded according to the International Classification of Diseases (ICD), 10th Revision.*

#### Data analysis

The analysis included demographic (age, country of birth, length of stay in Italy), socioeconomic (education, Italian language proficiency, marital status, housing, work status), administrative (legal status, SSN entitlement and registration), and health-related variables (prior health conditions, preferred healthcare service for consultation, diagnoses) for the SRH group. Descriptive statistics were used to summarize the data, with frequencies and percentages for categorical variables and means with standard deviations for continuous ones. Demographic, socioeconomic, and administrative characteristics were analysed for the entire SRH group, while clinical diagnoses excluded generic follow-up ICD codes. Subgroup comparisons (e.g., by country of origin or language proficiency) used chi-square tests for categorical and Student’s t-tests for continuous variables. The full dataset of women accessing EMERGENCY’s medical services was also examined to compare the frequency of inclusion in the SRH group, based on assigned diagnoses (as specified in Table 1), stratified by country of birth, work and legal status. To further strengthen the quantitative analysis, we conducted a multivariable logistic regression as a robustness check, with multiple imputation used as a sensitivity analysis; full details are reported in Annex 5. All analyses were conducted using STATA 19 (StataCorp, 2025), with an independent replication performed in R Studio (R Core Team, 2023). A significance threshold of *p* < 0.05 was applied.

### Qualitative study

The research team performed an in-depth qualitative study using semi-structured interviews to explore multiple stakeholders’ perspectives on barriers to SRH access in Milan, Italy. The methods follow the Consolidated Criteria for Reporting Qualitative Research (COREQ) (Tong *et al.*, 2007).

#### Research team and reflexivity

Three researchers (ES, AS, and ALC) with global health backgrounds and prior qualitative research experience and studies on access to care conducted the interviews. As members of a well-known NGO, they acknowledged their positionality, including potential influences of power dynamics or organizational trust. This may have fostered participant openness but could also have biased responses positively. When MW were familiar with the clinic, particular attention was paid to mitigate the impact of prior interactions on the interview process. Reflexivity was central throughout, with researchers remaining aware of how their roles and identities could shape interactions, data collection, and interpretation. Efforts focused on building trust, maintaining cultural and professional sensitivity, and centring diverse participant perspectives to reduce bias and ensure balanced representation.

#### Study design and participants’ recruitment

This qualitative study employed a phenomenological approach to explore participants’ lived experiences and how they interpret and assign meaning to their reality within a specific context (Tanwir *et al.*, 2021). MW were selected through convenience sampling from EMERGENCY’s Milan database, focusing on those who had accessed SRH services. Healthcare professionals from both public facilities and NGOs, as well as policymakers involved in SRH service organization in Milan, were recruited via purposive and snowball sampling.

Stakeholders were initially contacted by phone, informed of the study’s objectives, and, upon agreeing to participate, interviews were scheduled based on availability. Interviews were conducted in person whenever possible, either at the NGO offices or the interviewee’s workplace. Two were held online via Zoom [version: 5.10.4 (6592)], with privacy and confidentiality ensured.

#### Data collection

A semi-structured interview guide with leading and probing questions was developed in line with the study’s objectives. It focused on barriers faced by MW in accessing SRH in Milan, the strategies they use to overcome them, and their recommendations for improving access. The guide was adapted for healthcare workers (HCWs) and policymakers to gather their perspectives on barriers and system-level solutions. Piloted with researchers, it was then refined to ensure relevance across participant groups. Recruitment continued until data saturation was reached (Vasileiou *et al.*, 2018). Interviews were conducted in August 2024, averaging 50 minutes. All interviews were audio-recorded with consent and conducted in Italian, except for one, which was conducted in French with an interpreter. Notes were taken, and quoted text was translated for publication.

#### Data analysis and reporting

Interviews were de-identified and transcribed verbatim using Sonix Software [2023 Sonix, Inc.], with transcripts manually reviewed for completeness and quality. An inductive codebook was developed based on the study objectives and kept flexible to incorporate emerging codes.

Following a thematic analytic approach (Braun and Clarke, 2012) codes were organized into broader categories aligned with the socio-ecological framework (Stokols, 1996), itself rooted in Bronfenbrenner’s ecological systems theory (Bronfenbrenner, 1977). This allowed for a thorough analysis of how individual experiences and barriers to SRH services are shaped by structural and contextual aspects, emphasizing the interaction between individuals and their environment at various levels (individual, interpersonal, organizational, social, policy). The analysis was conducted in October 2024 by four researchers (ES, AS, DG, and ALC), with discrepancies resolved through discussion. Coding and analysis were performed using Atlas.ti [Version 24.1.1 (30840)].

## Results

**Figure 1.**
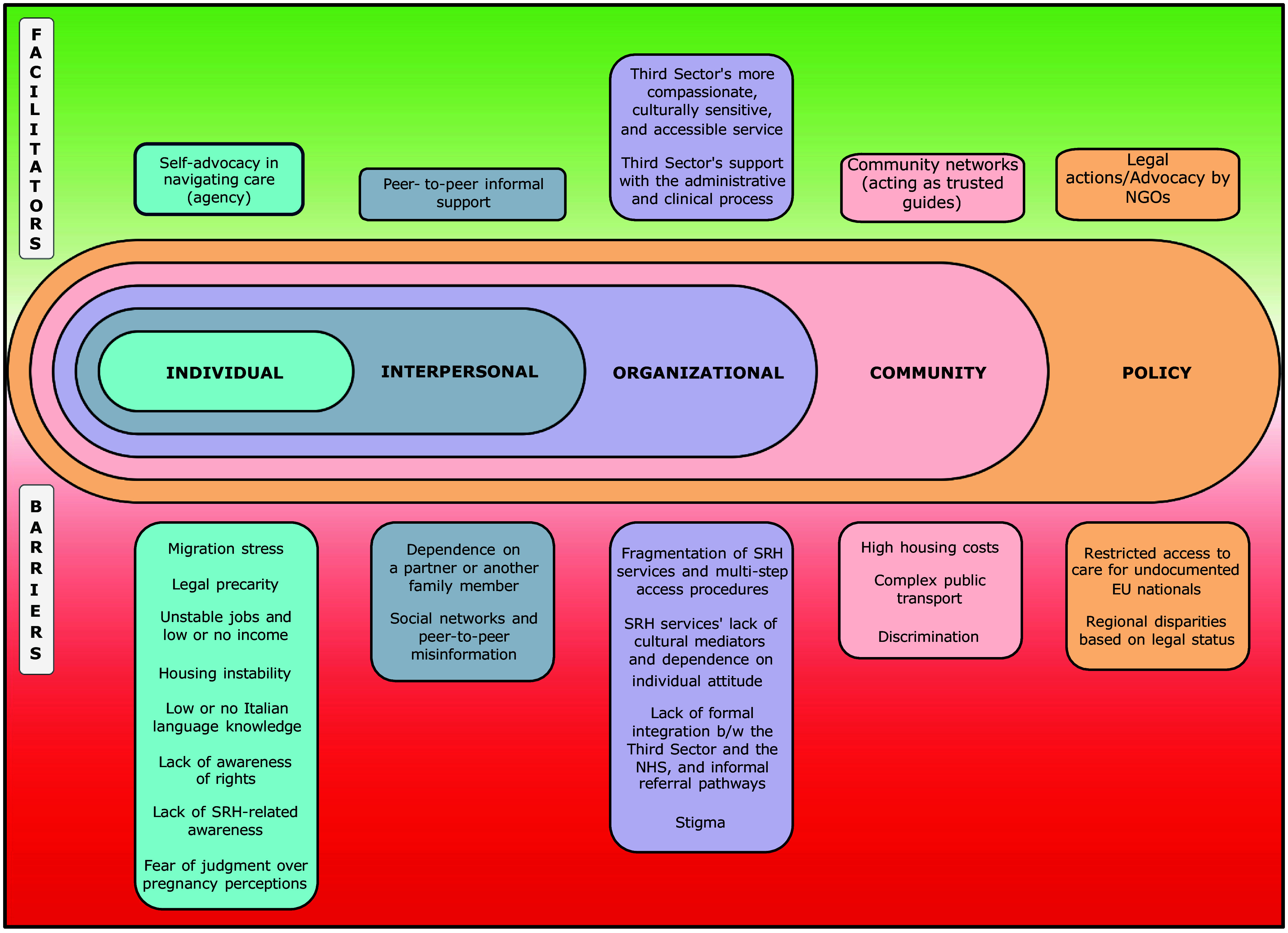
A graphical representation of the results based on the socio-ecological framework.

The main findings are summarized in Figure 1, which presents a graphical representation based on the socio-ecological framework.

### Quantitative analysis

Between July 1, 2017 and December 21, 2023, EMERGENCY’s primary care services recorded 57,830 accesses for 11,397 individuals in Milan. Among these, 31.6% (*n* = 3,601) identified as female. The largest group originated from Peru (24.2%, *n* = 873), followed by Romania (13%, *n* = 468), and Morocco (12,4%, *n* = 445), Italy (9.1%, *n* = 326), and Egypt (7%, *n* = 251).

Nearly one-third of the services provided were used by young females of reproductive age (18–34 years: 30.9%, *n* = 6527). Among medical consultations (*n* = 4838), the most frequent diagnoses were obstetrical-gynaecological conditions (13.8%, *n* = 655).

#### SRH group: demographic and socioeconomic profile

The SRH group included 431 medical consultations for 316 MW who accessed care for SRH-related diagnoses. Over half of the diagnoses assigned during these consultations were related to pregnancy confirmation or exclusion (56.6%, *n* = 244), followed by consultations for contraceptive management (17.9%, *n* = 77). Approximately one-third of the patients in the SRH group were born in Romania and nearly one-fifth in Morocco. Peru and Egypt were the third and fourth most common countries of birth. Seven women (2.2%, *n* = 7) were born in Italy and had a migratory background based on ethnicity and cultural ties. Annex 1 summarizes the SRH group characteristics as a whole and stratified by the four most frequently reported countries of birth.

In the SRH group, the mean age was ∼29 years. Romanian women were the youngest (mean age ∼26 years) and Peruvian patients were the oldest (mean age ∼31 years). Romanian patients had lived in Italy the longest (9.3 years on average, *p* < 0.05) and demonstrated sufficient knowledge of the Italian language (83%, *n* = 89, *p* < 0.05) compared to the women from the other countries (Table 1). In contrast, Egyptian women showed insufficient Italian language proficiency (87.5%, *n* = 28) and were more likely to live with a partner (84.4%, *n* = 27, *p* = 0.007). Over 70% of the women in the SRH-group reported having at least one child. Housing conditions were poor for nearly half of the group, with the worst conditions observed among women from Morocco (63.5%, *n* = 40) and Peru (54.2%, *n* = 26). Most women reported no income from work activities, particularly Egyptian women (96.7%, *n* = 30, *p* = 0.023).

Annex 2 provides the administrative profile of the SRH group, including legal status, SSN entitlement, and enrolment. Most patients were born outside the European Union (EU), and held either an Italian residence permit (30.7%, *n* = 97) or were undocumented migrants (29.1%, *n* = 92). Approximately 20% of patients were born in an EU country but lacked a regular residence permit.

Although over 40% of women in the SRH group were entitled to SSN care, about half of them were not enrolled due to administrative issues (e.g., lack of documentation, 37.3%, *n* = 25) or lack of awareness of their rights (32.8%, *n* = 22). Among those enrolled in the SSN, the primary reason for accessing EMERGENCY’s services was linguistic or logistical barriers (e.g., navigating procedures, appointment access) to using SSN services (47.7%, *n* = 31).

For women from EU countries not entitled to SSN enrolment (24.4%, *n* = 77), almost 90% lacked both health insurance and an ENI code. Similarly, nearly 75% of non-EU women not entitled to SSN enrolment did not hold the STP code, which is necessary for accessing SSN urgent and essential care. In most cases (59.7%, *n* = 40) this was due to a lack of perceived need or difficulties accessing the SSN.

In 20.9% of cases (*n* = 14), healthcare facilities failed to issue the STP code despite the patient accessing SSN services. Over half of the women not entitled to SSN enrolment sought care at EMERGENCY’s services based on advice from friends (58.1%, *n* = 97).

#### Socioeconomic and health-related features: associations

**Figure 2.**
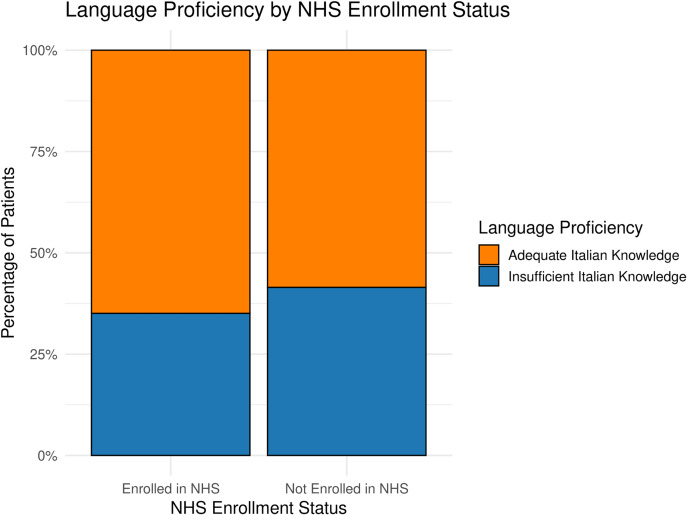
Association between Italian language proficiency and SSN enrolment among women entitled to register. No statistically significant difference was observed between women with sufficient and insufficient knowledge of Italian.

**Figure 3.**
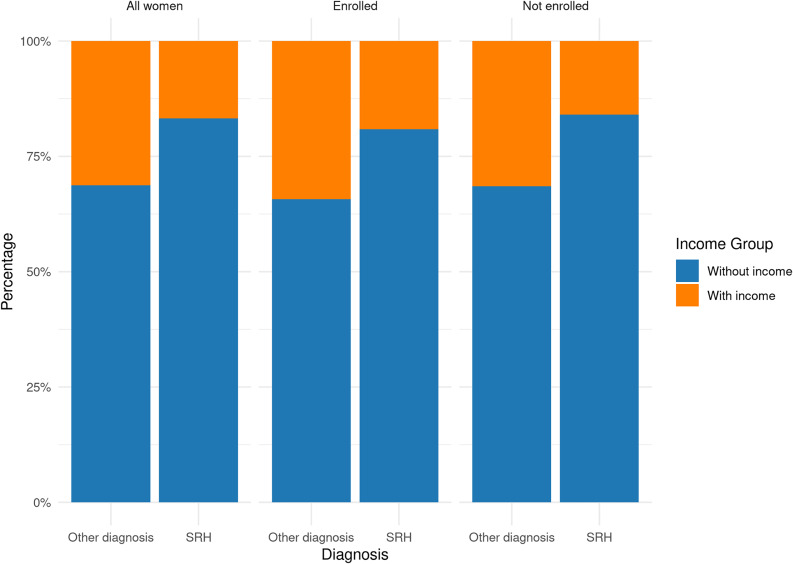
Association between income from labour activities and SRH-related diagnoses, stratified by SSN enrolment. Women with SRH-related diagnoses were significantly more likely to lack income compared to those with other diagnoses, and this pattern remained consistent across both enrolled and non-enrolled groups.

Within the SRH group, no statistically significant association was found between knowledge of the Italian language and SSN enrolment among patients entitled to SSN care (Figure 2).^2^ In the overall female population sample, women with an SRH-related diagnosis were significantly more likely to lack income from labour activities compared to those with other types of diagnoses (83.2% vs. 68.8%, *p* < 0.001). This association remained statistically significant when analyses were stratified by SSN enrolment status (Figure 3). Results of the multivariable logistic regression are reported in Annex 5, and were consistent with the descriptive and bivariate analyses, confirming the robustness of the observed associations (van Buuren and Groothuis-Oudshoorn, 2011; Westreich and Greenland, 2013).

### Qualitative analysis

Interviews were conducted with 29 participants, including eleven MW, eight HCWs from governmental facilities, four NGO representatives, and six policymakers. A breakdown of all respondents’ characteristics is provided in Annex 3. Findings will be presented through the socio-ecological framework highlighting barriers and facilitators at individual, interpersonal, organizational, societal, and policy levels.

#### Individual factors

MW in Milan navigate a complex web of legal, financial, and administrative barriers, compounded by social and cultural expectations and precarious SDH. Many interviewed women reported bearing caregiving responsibilities, being economically dependent, and working in low-paid, unstable jobs with inflexible schedules. These intertwined challenges constrain their ability to seek care and access SRH services, often forcing them to choose between basic necessities and medical care. Some require a male companion for medical appointments or must defer SRH-related decisions to their families, compromising privacy. Many participants were unaware of entitlements like the STP card, or struggle with its complex processes.

MW often perceive pregnancy differently from the Western norm, which they view as overly medicalized, and tend to adopt a more ‘fatalistic’ approach, placing less emphasis on prenatal check-ups or frequently missing these appointments. In other cases, lack of pregnancy-related awareness was observed.

*“When they said I needed help for my pregnancy, I didn’t understand anything at all about all these check-ups—I knew nothing about them” (MW 03)*

MW’s mental health needs are widely overlooked by healthcare professionals, with MW citing isolation, migration stress, and lack of culturally sensitive psychological support as major concerns.

*“Mental health services for migrants? A second-rate problem for second-class people.” (Healthcare worker, NGO)*

Postpartum and neonatal care are also difficult to access without proper documentation according to many MW.

#### Interpersonal factors

Social networks may serve as key facilitators of access to care, with many MW relying on community connections, especially within Latin American groups, which provide informal support and guidance. However, these networks were also reported to occasionally spread misinformation. In the absence of formal language support, MW often depend on family members for interpretation during medical visits, limiting autonomy and potentially compromising communication accuracy. While some communities have robust internal support networks, others remain comparatively isolated and often unaware of available services, particularly those without established networks or whose husbands work long hours.

*“I have a group of Latinas, with whom I would always consult about which hospital they recommended I go to. I found a friend in this group, she was also pregnant, and in some way, she helped me find everything.” (MW)*

#### Organizational factors

For MW, accessing SRH services in Milan often involves multi-step procedures, delays, and frustration, with variations in service quality depending on individual staff attitudes and knowledge. Some HCWs also report discrepancies in the services provided to the same patients across different public health facilities. Even when access is achieved, MW’s right to health is not fully guaranteed. For example, structural discrimination, racism, and fear of judgement can deter MW from engaging with these services. Many MW, in fact, reported that HCWs may lack cultural competence, reinforcing negative stereotypes and biases that erode MW’s trust in the system.

*“When these people are here, you can’t pretend not to see them. Either you give them access to services or you don’t. But this ambiguity, where so many people in Milan live without access to social and healthcare services because they don’t have documents, significantly impacts services. It’s not that the office is missing, it’s that our right mindset is lacking” (SSN worker)*

The limited availability of a primary care system in Milan leads to a reliance on emergency departments for non-urgent needs, further straining the SSN, which is already burdened by HCW shortages and long waiting times. For example, some MW reported turning to emergency departments as a faster way to obtain a pregnancy certificate, reflecting limited awareness of alternative options and the perceived simplicity of this pathway for administrative procedures.

MW frequently turn to NGOs for culturally sensitive, patient-centred care, often perceiving NGO providers as more compassionate and accessible than institutional settings. According to a policymaker, NGOs address gaps in the SSN by providing free healthcare, legal advocacy, and culturally tailored mental health support. However, these services are often unable to provide the sustainability and continuity needed for comprehensive, long-term support, particularly in the realm of prevention. Moreover, these services lack formal integration with the SSN, limiting care continuity. Some individuals rely exclusively on NGOs to access care, highlighting systemic inequalities and perpetuating a dual healthcare system. Referral pathways from public hospitals to NGOs are often informal, relying on personal connections rather than institutional protocols. Within this fragmented landscape, cultural mediators emerged as essential figures. Their role, central to many NGO-led interventions, extends far beyond linguistic translation. By drawing on their lived experience and cultural knowledge, they help build trust, personalize care, and support patients through complex administrative and clinical processes. As described by one interviewee they are *agents of empowerment*, who not only deliver health education and facilitate navigation through the health system, but also proactively reach out to individuals who might otherwise remain invisible to services. Their contribution is particularly crucial in sustaining continuity of care across fragmented service landscapes, especially where formal SSN integration is lacking.

NGOs’ broader advocacy for migrant rights was also noted as a key facilitator of access. This includes informing individuals about their entitlements, pursuing legal actions in response to systemic exclusion, and applying pressure on local authorities to resolve cases before they escalate to court. However, these legal proceedings are often prolonged and costly, leading some patients to forgo legal action and pay to access immediate care.

#### Societal factors

Milan’s urban layout, high housing costs, and complex public transport system create logistical barriers to SRH care. While central facilities benefit from strong transit links, decentralization forces MW in peripheral areas to undertake long, expensive journeys to access essential services. Mobile clinics, while helpful, only offer basic care and do not address specialized SRH needs.

Additionally, relocating administrative offices farther from main facilities increases travel burdens and adds steps to accessing necessary resources, discouraging patients from returning or completing required procedures.

#### Policy factors

Several interviewees observed a shift away from universal healthcare access in Italy, despite constitutional and legal guarantees of equity and non-discrimination. Practical implementation often falls short and shows inconsistencies, especially for vulnerable groups like MW. For example, Italian law supports healthcare access for pregnant women, yet, in practice, access is highly restricted and inconsistently applied and often contingent upon having valid documentation. A paradox emerged clearly during the interviews, particularly for certain EU citizens (i.e., Romanians) who lack coverage in their home countries and an official recognition as residents in Milan, leaving them in a *legal limbo* that is exacerbated by restrictive regional policies (i.e., the lack of ENI code in Lombardy). This may force them to pay for SRH services. In the view of some policymakers, authorities rely on NGOs to fill healthcare gaps, rather than structurally integrating services, reflecting political choices that undermine universal access.

#### The ‘invisible’ populations

Policymakers and health professionals expressed concern about MW who remain entirely excluded from both the SSN and NGO support, often referred to as *invisible*. These individuals are disconnected from local networks, and avoid SRH care due to more pressing issues, distrust, misinformation, cultural barriers, or complex legal statuses. Fear of legal repercussions, such as deportation or losing child custody, further deters them from seeking help, even from trusted NGOs. Consequently, these individuals only access the healthcare system during acute SRH problems, leading to worse outcomes and increased mental health burden. Some interviewees also noted that this situation poses broader societal challenges, increasing costs by focusing on managing acute complications rather than preventing them.

*“These women only exist when they fall ill, otherwise, they are invisible.” (NGO worker)*

The COVID-19 pandemic exacerbated invisibility, with increasing resource competition and economic precarity, making MW even more vulnerable. In the opinion of many interviewees, proactive outreach and integration of community-based care models were important during the pandemic and are critical to addressing these gaps.

## Discussion

Using a mixed-methods approach, this study examines the characteristics of MW accessing an NGO outpatient clinic for SRH needs and the barriers influencing their access to SRH care within the Italian SSN in Milan. SRH is increasingly pivotal in the health experiences of MW, given their reproductive age profile and documented worse outcomes compared to host populations (World Health Organization, 2012; UNFPA, 2019). Our findings, consistent with other Milan-based services (NAGA, 2024), highlight the high demand for SRH services and the crucial role of NGOs in filling SSN gaps. Additionally, the study’s findings underscore the layered and systemic challenges, shaped by social determinants, that influence SRH access and create a cumulative disadvantage for MW.

Economic vulnerability significantly influenced SRH access. Women with SRH-related diagnoses were more likely to lack income, reflecting the link between financial precariousness, housing instability, and forgone care (Keygnaert *et al.*, 2016; Funge *et al.*, 2020; Feldman, 2021; Nellums *et al.*, 2021; Gogishvili *et al.*, 2021). This association remained significant after adjusting for SSN enrolment, showing that formal entitlement alone does not remove financial barriers, as indirect costs like transport and medication persist. Similar ‘triple jeopardy’ dynamics have been described elsewhere. (Nellums *et al.*, 2021).

Beyond financial barriers, MW often struggle to navigate the SSN due to limited knowledge of entitlements and complex administrative requirements (Higginbottom *et al.*, 2016; Sami *et al.*, 2019). Interpersonal factors, such as family and partner dynamics can also limit autonomy in their health-seeking behaviours. Interestingly, language barriers, despite being widely recognized as significant stressors (Jiménez-Lasserrotte *et al.*, 2020; Egli-Gany *et al.*, 2021; Machado *et al.*, 2022; Barrio-Ruiz *et al.*, 2024) as well as length of residence, did not significantly influence SRH access in our study. However, qualitative insights showed their relevance: mediators mitigated linguistic barriers, while longer residence did not overcome structural exclusions. Overall, this multifaceted set of challenges creates a labyrinthine system that complicates access to healthcare, emphasizing that equitable care requires not just formal SSN enrolment but services that are approachable, culturally sensitive, and designed for active engagement.

On an organizational level, the fragmentation of SRH services in Milan poses significant challenges. MW often navigate multi-step procedures, long waiting times, and inconsistencies in service quality, with patients receiving different treatments, varying interpretations of protocols, and inconsistent application of exemptions across public facilities. Stigma, discrimination, and fear of judgement from HCWs were noted as deterrents, consistent with prior studies (Bollini *et al.*, 2007; Castañeda, 2008; Sanò *et al.*, 2024). Cultural mediators emerged as key facilitators, providing navigation and culturally informed support, yet their availability and expansion are uneven due to budgetary priorities and fragmented governance, and often limited to NGOs. Expanding structured, sustainable cultural mediation services within the SSN is thus critical for equitable access (Ciribuco and Federici, 2024). As a result, many MW turn to NGOs, valuing their approachability, mental health support, and culturally tailored services, but their services often lack sustainability and integration, creating a dual system that perpetuates inequities (Peralta-Gallego *et al.*, 2018; Barkensjö *et al.*, 2018; Weller *et al.*, 2019; Linke *et al.*, 2019; Tasa *et al.*, 2021; Gogishvili *et al.*, 2021; Funge *et al.*, 2022). Formal NGO-SSN collaboration could improve care integration and continuity of care and reduce inefficiencies (Rosso *et al.*, 2023; Orru *et al.*, 2021). If adequately coordinated, NGOs could support the SSN in reaching the most marginalized individuals, guiding them through a more straightforward and comprehensive care pathway, enhancing preventive approaches, and addressing the broader SDH (Parotto *et al.*, 2023). Policy reforms ensuring uniform healthcare access for MW, regardless of administrative status, are urgently needed. For example, Romanian women in Lombardy remain excluded from SSN enrolment due to the lack of ENI code adoption, a legal gap that undermines universal health coverage (NAGA, 2024). While legal actions have sometimes addressed these gaps (Quotidianosanità.it, 2021; Ansa.it, 2021), they remain resource-intensive and often unsustainable. Other Italian regions, however, have adopted more inclusive practices (e.g. implementing ENI codes for European citizens), showing that feasible reforms are possible within the national framework if regional policies align with universal access principles.

The mixed-methods design revealed convergences and nuances between quantitative and qualitative data. Lack of income was a key predictor of SRH consultations, consistent with narratives of financial dependence, precarious work, and difficult trade-offs between basic needs and healthcare. Higher odds among Romanian, Moroccan, and Peruvian women were enriched by qualitative insights of *legal limbo*, poor housing, and unstable employment. In contrast, variables non-significant in the regression, language and length of stay, were highlighted in interviews as barriers mitigated by mediators or exacerbated by persistent exclusions. This triangulation shows how legal and socioeconomic vulnerabilities structure SRH access, while lived dynamics shape when and how women engage with care.

An intersectional lens (Crenshaw, 1991), further illuminates how gender, migration status, and socioeconomic vulnerability reinforce one another (Hankivsky *et al.*, 2010; Equihealth, 2014; Kapilashrami and Hankivsky, 2018; Trentin *et al.*, 2025). Caregivers, for instance, face challenges due to inflexible schedules and inability to leave precarious jobs, often concentrated in informal sectors such as domestic work, reflecting gendered expectations. Others must rely on male companions or defer decisions, reflecting entrenched gender dynamics in healthcare access. Migrant status further compounds these barriers, as reluctance to engage with SRH services is linked to racism, discrimination, and fear of legal repercussions. Taken together, these findings show how gender, migration status, and socioeconomic vulnerability mutually reinforce one another and produce compounded disadvantages in accessing SRH care.

Finally, our analysis reveals the vital role of communities and social networks as facilitators of SRH access. Over half of MW ineligible for SSN enrolment sought care based on peer recommendations, echoing interview findings on the importance of community support This suggests that community-based approaches could enhance SRH access for MW in Milan, aligning with existing evidence on their effectiveness in improving SRH outcomes, particularly for migrant and hard-to-reach populations (World Health Organization, 2015; Lorente *et al.*, 2021; Mosnier *et al.,* 2024). Building on this dynamic, community health workers (CHWs) could extend outreach, education, and navigation support, especially for ‘invisible’ populations disconnected from both SSN and NGOs. Evidence from other settings shows that CHWs expand SRH access and improve health outcomes (Lorente *et al.*, 2021; Mosnier *et al.,* 2024). The COVID-19 pandemic underscored their value in reaching isolated populations and the need for more resilient, inclusive systems (Trentin *et al.*, 2024). Investing in CHW-led models could help bridge gaps, enhance preventive care, and facilitate integration into the SSN, benefitting MW and the broader public health landscape, particularly during crises and emergencies.

## Strengths and limitations

The mixed-methods approach is a key strength of this study, offering a comprehensive understanding of the barriers and facilitators shaping MW’s access to SRH care. By integrating quantitative and qualitative data, the analysis captures both numerical trends and lived experiences, contributing to a more nuanced and contextually grounded perspective. The use of the socio-ecological model adds conceptual depth, guiding the analysis across multiple levels and supporting the development of integrated, multi-layered recommendations (see Table 2). However, the study did not fully apply an intersectional approach, and future research should explicitly examine how overlapping identities and vulnerabilities shape SRH access among MW.

**Table 2.** Recommended strategies derived from study findings

Socio-ecological level	Recommended strategies
*Individual*	● Increase health literacy through targeted education campaigns ● Provide culturally sensitive mental health support
*Interpersonal*	● Strengthen community networks through community health worker-based strategies
*Organizational*	● Formalize collaboration of NGO services with the SSN ● Enhance primary care infrastructure at the peripheral level ● Expand the role and presence of cultural mediators
*Societal*	● Implement proactive outreach programmes for marginalized populations ● Address systemic biases through HCW training
*Policy*	● Reform regional policies to ensure universal access ● Eliminate legal and administrative barriers ● Formally incorporate NGO services into public health frameworks.

With regard to the quantitative data, this was originally collected for clinical purposes, so some variables may be incomplete or inconsistently recorded, impacting the depth of the analysis and its generalizability. The study also focused on MW who had already accessed NGO services, excluding harder-to-reach populations. Nevertheless, qualitative insights help explain why some individuals remain ‘off-the-radar’, offering valuable guidance for strategies to engage marginalized groups. Although the analysis explored associations between sociodemographic factors and SRH service use, it excluded non-clinical services like cultural mediation, limiting insight into their uptake. Missing data in some variables further reduced sample representativeness, limiting certain analyses. The qualitative component, though based on a relatively small sample, benefited from the participant diversity, providing rich perspectives that strengthened the overall findings. While additional participants could have added nuances (Dey, 1999), the recurrence of key themes justified concluding data collection.

## Conclusion

This study highlights systemic, organizational, and policy barriers shaping MW’s access to SRH care in Milan. Financial instability, administrative challenges, and fragmented services create significant hurdles, while NGOs play a crucial but unsustainable role in filling gaps. Improving access to SRH care for MW is not only a matter of equity but also a critical public health priority, given its profound impact on societal well-being, the health of future generations, and economic stability. Expanding cultural mediation, formalizing NGO-SSN collaboration, and investing in community-based approaches, including CHWs, are key steps toward ensuring equitable SRH access for MW. Policy reforms addressing regional disparities and bureaucratic inconsistencies are essential to uphold the right to healthcare for all MW, aligning with Italy’s universal healthcare principles.

## Supporting information

Marro et al. supplementary material 1 Marro et al. supplementary material

Marro et al. supplementary material 2 Marro et al. supplementary material

Marro et al. supplementary material 3 Marro et al. supplementary material

Marro et al. supplementary material 4 Marro et al. supplementary material

Marro et al. supplementary material 5 Marro et al. supplementary material

## Data Availability

The data supporting this study are available from the corresponding author upon reasonable request.

## Footnotes

1 In the Italian health system, undocumented non-EU nationals temporarily residing in Italy are assigned the STP (Straniero Temporaneamente Presente) code for emergency and essential healthcare, while undocumented EU nationals receive the ENI (Europeo Non Iscritto) code, providing access to essential healthcare services. Both codes are short-term, yet renewable under specific conditions (Testo Unico Immigrazione, 1998).

2 Language proficiency is routinely assessed by EMERGENCY’s cultural mediators during the first interaction with patients. Adequate knowledge of Italian language is categorized as optimal, average, sufficient, or Italian identified as the mother tongue. Insufficient knowledge of the Italian language is categorized as scarce or nonexistent.

## Supplementary material

The supplementary material for this article can be found at https://doi.org/10.1017/S1463423626100954.
