# Supplementary material for: Navigating barriers and building solutions: a mixed-methods study on sexual and reproductive healthcare for migrant women in Milan
Source: Prim Health Care Res Dev. 2026 Feb 27;27:e29. doi: 10.1017/S1463423626100954 (PMC12964159; doi:10.1017/S1463423626100954)
Supplement: Marro et al. supplementary material 1 — Marro et al. supplementary material [file S1463423626100954sup001.docx]

|  | **Romania** | **Morocco** | **Peru** | **Egypt** | **Overall population**  **(SRH group)** |
| --- | --- | --- | --- | --- | --- |
| **Total, n (%)** | 107 (33.9) | 63 (19.9) | 48 (15.1) | 32 (10.1) | 316 (100.0) |
| **Age, mean (SD)** | 26.3 (7.1) | 29.7 (5.6) | 31.4 (7.3) | 30.1 (7.3) | 28.9 (7.4) |
| **Reason for migrating, n (%)** |  |  |  |  |  |
| *Economic reasons* | 80 (74.8) | 36 (57.1) | 29 (60.4) | 5 (15.6) | 172 (54.4) |
| *War, persecution* | 0 | 3 (4.8) | 2 (4.2) | 0 | 13 (4.1) |
| *Familiar and ethnic problems* | 1 (0.9) | 5 (7.9) | 3 (6.3) | 1 (3.1) | 14 (4.4) |
| *Family reunification* | 25 (23.4) | 15 (23.8) | 11 (22.9) | 24 (75) | 89 (28.2) |
| *Other* | 1 (0.9) | 4 (6.4) | 2 (4.2) | 1 (3.1) | 12 (3.8) |
| *Missing data* | 0 | 0 | 1 (2.1) | 1 (3.1) | 16 (5.1) |
| **Length of stay in Italy, mean nr of years (SD)**  *(data missing for 18 women: 1 from Egypt, 1 from Peru and 1 from Morocco, none from Romania)* | 9.3 (6.1) | 6.7 (7.8) | 2.5 (5.8) | 5.5 (7.8) | 8.4 (10.2) |
| **Permanence in Italy, n (%)** | | | | | |
| *< 3 years* | 25 (23.4) | 30 (47.6) | 39 (81.2) | 16 (50.0) | 136 (43.0) |
| *> 3 years* | 82 (76.6) | 32 (50.8) | 8 (16.7) | 15 (46.9) | 162 (51.3) |
| *Missing data* | 0 | 1 (1.6) | 1 (2.1) | 1 (3.1) | 18 (5.7) |
| **Knowledge of Italian language, n (%)*** | | | | | |
| *Sufficient* | 89 (83.2) | 29 (46.0) | 23 (47.9) | 4 (12.5) | 177 (56.0) |
| *Insufficient* | 18 (16.8) | 34 (54.0) | 25 (52.1) | 28 (87.5) | 131 (41.5) |
| *Missing data* | 0 | 0 | 0 | 0 | 8 (2.5) |
| **Marital status, n (%) #** | | | | | |
| *Co-residing partner* | 79 (73.8) | 25 (39.7) | 22 (45.8) | 27 (84.4) | 185 (58.5) |
| *Non-Co-residing partner* | 27 (25.2) | 38 (60.3) | 26 (54.2) | 5 (15.6) | 122 (38.6) |
| *Missing data* | 1 (0.9) | 0 | 0 | 0 | 9 (2.8) |
| **Children, n (%)** | | | | | |
| *None* | 17 (15.9) | 23 (36.5) | 9 (18.8) | 5 (15.6) | 79 (25.0) |
| *One or more* | 90 (84.1) | 40 (63.5) | 39 (81.2) | 27 (84.4) | 229 (72.5) |
| *Missing data* | 0 | 0 | 0 | 0 | 8 (2.5) |
| **Housing conditions, n (%)°** | | | | | |
| *Poor* | 51 (47.7) | 40 (63.5) | 26 (54.2) | 13 (40.6) | 154 (48.7) |
| *Stable* | 50 (46.7) | 19 (30.2) | 21 (43.7) | 18 (56.2) | 141 (44.6) |
| *Missing data* | 6 (5.6) | 4 (6.3) | 1 (2.1) | 1 (3.1) | 21 (6.7) |
| **Work status, n (%) ¶ ^** |  |  |  |  |  |
| *Unemployed* | 61 (57.0) | 35 (55.6) | 27 (56.2) | 13 (40.6) | 172 (54.4) |
| *Employed* | 10 (9.35) | 10 (15.9) | 18 (37.5) | 1 (3.1) | 53 (16.8) |
| *Not in the labor force* | 28 (26.2) | 13 (20.6) | 2 (4.2) | 17 (53.1) | 67 (21.2) |
| *Missing data* | 8 (7.5) | 5 (7.9) | 1 (2.0) | 1 (3.1) | 24 (7.6) |
| **Income, n (%)§** | | | | | |
| *No* | 89 (89.9) | 48 (82.8) | 29 (61.7) | 30 (96.7) | 239 (75.6) |
| *Yes* | 10 (10.1) | 10 (17.2) | 18 (38.3) | 1 (3.2) | 53 (16.8) |
| *Missing data* | 8 (7.5) | 5 (7.9) | 1 (2) | 1 (3.1) | 24 (7.6) |

**Annex 1: SRH group: demographic e socioeconomic features**

**Sufficient knowledge of Italian language: optimal, on average, sufficient or Italian stated as mother tongue. Insufficient knowledge of Italian language: scarce or null.*

*# Co-residing partner: women married to a Non-EU or EU Spouse in Italy, cohabitant or who underwent common-law marriage. Non-co-residing partner: single, never married, married to a spouse Abroad or separated/divorced women or widows).*

*° Poor housing conditions: homelessness, unsafe or inappropriate housing conditions. Stable housing conditions: rent, hosted by employers, homeowner (council house or not).*

¶ *Unemployed: unemployed/waiting for employment, unoccupied.*

*Employed: elderly care, commercial activity, construction, catering/hotels, transport, other activity.*

*Not in the labor force: housewife, student, retiree.*

*^ One Peruvian patient declared to be unemployed at the first two visits, while at the third visit she reported to have found a job. Data in the table is limited to the work status declared at the first visit.*

*§ Income was defined as limited to revenues from work activities. It was assessed according to work status as “no” for unemployed or inactive women, housewives and students and “yes” for the remaining women with a known work status.*
