# Supplementary material for: Navigating barriers and building solutions: a mixed-methods study on sexual and reproductive healthcare for migrant women in Milan
Source: Prim Health Care Res Dev. 2026 Feb 27;27:e29. doi: 10.1017/S1463423626100954 (PMC12964159; doi:10.1017/S1463423626100954)
Supplement: Marro et al. supplementary material 2 — Marro et al. supplementary material [file S1463423626100954sup002.docx]

**Annex 2 SRH group: administrative features and access to SSN services**

|  | **Romania** | **Morocco** | **Peru** | **Egypt** | **Overall population**  **(SRH group)** |
| --- | --- | --- | --- | --- | --- |
| **Total, n (%)** | 107 (33.9) | 63 (19.9) | 48 (15.1) | 32 (10.1) | 316 (100.0) |
| **Legal status, n (%)** | | | | | |
| *Non EU with permit* | - | 33 (52.3) | 14 (29.2) | 27 (84.4) | 97 (30.7) |
| *Non EU without permit* | - | 28 (44.4) | 34 (70.8) | 3 (9.3) | 92 (29.1) |
| *EU non regular* | 61 (57.0) | - | - | - | 65 (20.6) |
| *EU regular* | 41 (38.3) | - | - | - | 43 (13.6) |
| *Italian* | 1 (0.9) | 1 (1.6) | - | 1 (3.12) | 6 (1.9) |
| **Entitlement to be enrolled in the SSN, n (%)** | | | | | |
| *Entitled* | 34 (31.8) | 34 (54.0) | 11 (22.9) | 27 (84.3) | 132 (41.8) |
| *Enrolled* | 18 (52.9) | 16 (47.0) | 2 (18.2) | 20 (74.0) | 65 (49.2) |
| *Not enrolled* | 16 (47.0) | 18 (52.9) | 9 (81.8) | 7 (25.9) | 67 (50.8) |
| *Not-Entitled non EU* | - | 27 (42.9) | 35 (72.9) | 4 (12.5) | 90 (28.5) |
| *Not-Entitled EU* | 70 (65.4) | - | - | - | 77 (24.4) |
| **Healthcare services previously accessed**  *(data on 193 patients who declared to have previously experienced a health problem. Among those, 69 were from Romania, 44 from Morocco, 21 from Peru and 21 from Egypt)* | | | | | |
| *Emergency Room* | 39 (56.5) | 16 (36.4) | 7 (33.3) | 6 (28.6) | 80 (41.5) |
| *Outpatient services within the SSN* | 2 (2.9) | 1 (2.3) | 0 | 0 | 5 (2.6) |
| *Other NGO/volunteer-based services* | 5 (7.3) | 4 (9.1) | 6 (28.6) | 1 (4.8) | 21 (10.9) |
| *General Practitioner* | 11 (15.9) | 14 (31.8) | 1 (4.8) | 11 (52.4) | 49 (25.4) |
| *Pharmacy* | 3 (4.3) | 1 (2.3) | 2 (9.5) | 1 (4.8) | 9 (4.7) |
| *Other* | 4 (5.8) | 6 (13.6) | 3 (14.3) | 3 (14.3) | 20 (10.4) |
| *Missing data* | 5 (7.3) | 2 (4.6) | 2 (9.5) | 0 | 9 (4.7) |
