# Supplementary material for: Navigating barriers and building solutions: a mixed-methods study on sexual and reproductive healthcare for migrant women in Milan
Source: Prim Health Care Res Dev. 2026 Feb 27;27:e29. doi: 10.1017/S1463423626100954 (PMC12964159; doi:10.1017/S1463423626100954)
Supplement: Marro et al. supplementary material 3 — Marro et al. supplementary material [file S1463423626100954sup003.docx]

#### **Annex 3: Breakdown of interviewees (n=29)**

#### **Migrant Women**

| **ID** | **Age** | **Country of Origin** | **Year of Arrival in Italy** | **Legal Status** | **Gender** |
| --- | --- | --- | --- | --- | --- |
| 1 | 40 | Morocco | 2001 | Legally resident | F |
| 2 | 25 | Romania | 2019 | Not legally resident | F |
| 3 | 36 | Albania | 2014 | Legally resident | F |
| 4 | 27 | Peru | 2024 | Legally resident | F |
| 5 | 21 | Tunisia | 2023 | Legally resident | F |
| 6 | 32 | Romania | 2012 | Not legally resident | F |
| 7 | 35 | Peru | 2023 | Legally resident | F |
| 8 | 23 | Peru | 2018 | Legally resident | F |
| 9 | 30 | Senegal | 2023 | Not legally resident | F |
| 10 | 30 | Peru | 2022 | Legally resident | F |
| 11 | 36 | Albania | 2023 | Not legally resident | F |

#### **Healthcare Workers (HCWs)**

| **ID** | **Interviewee’s Role** | **Gender** |
| --- | --- | --- |
| 1 | Coordinator of an obstetric outpatient clinic | F |
| 2 | Psychologist in a high-risk pregnancy unit | F |
| 3 | Cultural mediator for healthcare orientation in a public family health center | F |
| 4 | Administrative staff member in a public family health center | F |
| 5 | Social worker supporting access to termination of pregnancy services | F |
| 6 | Gynecologist in a public family health center | F |
| 7 | Senior gynecologist in charge of termination of pregnancy services | F |
| 8 | Hospital-based gynecologist | M |

#### **NGO Workers**

| **ID** | **Respondent's Role** | **Gender** |
| --- | --- | --- |
| 1 | Legal support for migrants at an NGO in Milan | F |
| 2 | Coordinator of outreach interventions for Roma and Sinti communities | F |
| 3 | Physician at a primary care clinic run by an NGO in Milan | F |
| 4 | Nurse for a migrant reception project | F |

#### **Policymakers**

| **ID** | **Respondent's Role** | **Gender** |
| --- | --- | --- |
| 1 | Former Regional Councilor | M |
| 2 | Former Regional Councilor | F |
| 3 | Regional coordinator of migrant reception centre | F |
| 4 | Municipal Councilor | F |
| 5 | Anti-trafficking project officer for local municipality in Milan area | F |
| 6 | Regional Council Member | M |
