# Supplementary material for: Navigating barriers and building solutions: a mixed-methods study on sexual and reproductive healthcare for migrant women in Milan
Source: Prim Health Care Res Dev. 2026 Feb 27;27:e29. doi: 10.1017/S1463423626100954 (PMC12964159; doi:10.1017/S1463423626100954)
Supplement: Marro et al. supplementary material 4 — Marro et al. supplementary material [file S1463423626100954sup004.docx]

**Annex 4: Interview guide and codebook**

Interview guide

**INTERVIEW WITH MW**

**Outline a profile of the interviewees**

How old are you? When did you arrive in Italy? How do you feel here? What do you do for a living/what is your current job? (Who do you live with?)

**Explore knowledge/expectations about the rights guaranteed by the SSN**

What did you know about the SSN when you arrived in Italy? What was your idea of healthcare? What did you expect/know about the Italian healthcare system?

In your opinion, what rights do you have in Italy regarding the protection of your health? What does the Italian state offer you?

How/where did you obtain/find information about your rights?

**Understand which SSN services they use/know about**

The first time you needed medical care, what did you do? Who/where did you turn to? How did it go? Were you able to resolve your problem?

Have you used other services (clinics, doctors, volunteer associations...)? Which ones? How did you find out about their existence?

**Identify barriers to accessing and using services**

What difficulties/barriers did you encounter when trying to access/use the services you mentioned?

**Identify potential facilitators for access and use of services**

What elements/factors/people helped you access/use the services you mentioned, to meet your health needs?

**Identify barriers encountered in the SSN during pregnancy/IVG**

We are talking with you because we saw that you arrived at XXX. Why? How did you reach the association?

Before arriving at XXX, had you turned to any other SSN service? What difficulties/barriers/obstacles did you encounter (negative aspects/things you did not like or that made you feel uncomfortable)?

**Explore potential facilitators encountered in the SSN during pregnancy/abortions**

What was useful/helped you?

What aspects/elements/situations/people were helpful?

**Learn about their proposals and suggestions to facilitate access to services and their use**

In your opinion, what could be done to help other women in your situation?

Do you know other women in a similar/worse/better condition?

**INTERVIEW WITH HEALTHCARE PROVIDERS (both from NGOs-associations and the public system, counseling centers, etc.)**

**Outline a profile of the interviewee and their work with migrants**

What is your profession (doctor, nurse...)?

What type of service do you work in?

Which migrant communities do you deal with in your work?

Do you perceive differences in their health needs and necessities?

How was it emotionally and practically for you to take care of such a delicate situation?

Has working with migrants influenced your perspective as a healthcare provider?

**Explore the perception that healthcare staff have regarding the barriers and facilitators that women encounter in accessing healthcare**

Have you dealt with pregnant women or those who have recently given birth?

In your opinion, do pregnant women or those with gynecological issues who come to you encounter difficulties/barriers in accessing and using services? Which ones? Can you provide examples?

What factors, on the other hand, facilitate their access to services?

Do you notice differences in how Italian women approach these services?

How does patient care conclude? What happens next? Are they properly directed within the SSN, or do you believe there are difficulties of any kind?

**Identify the barriers that healthcare personnel encounter in providing care**

What challenges and difficulties do you perceive in assisting these women?

Are there aspects of your practice that are less familiar or less well-known?

**Identify factors that facilitate/help in the provision of healthcare**

In your experience, what has helped you in these situations?

What factors help you find an effective solution/support in difficult moments?

**Understand the provider's training and the presence of support from their institution**

What kind of training have you received/do you receive to work with migrants?

How do the organizational conditions/practices of the service/institution where you work influence the assistance provided to migrant women?

**Learn about their proposals/suggestions**

What aspects would you improve, or what would you need to improve the care provided to women?

**INTERVIEWS WITH INSTITUTIONAL PROFILES**

**Outline a profile of the interviewee**

What is your job? How long have you held this position?

What are your areas of responsibility?

Is there a working group that deals with this issue? If so, who is part of it? What stakeholders are involved?

**Identify the level of interconnection with local realities**

Do you interact/collaborate with volunteer associations/NGOs that assist migrants? If so, which associations and on what aspects? If not, why?

If the work of private social organizations did not exist, would the SSN be able to accommodate these people?

Do you believe that the SSN, in theory, works, or are there regulatory gaps to be filled?

**Understand the awareness level of difficulties in assisting migrants**

What do you think are the difficulties migrants face in accessing healthcare services? Specifically, do you think there are particular difficulties for women?

What do you think are the difficulties that healthcare and social workers face in assisting the migrant population?

**Understand the priority and urgency assigned to this issue**

What are the most urgent issues to be resolved, in your opinion?

What priority and urgency do you assign to these topics?

**Identify ongoing actions aimed at finding solutions**

Are there any projects currently underway or planned to facilitate the integration of these people into the SSN, considering that it is universalistic?

What possible solutions/improvements do you propose?

Codebook

General information

*Women*

1. Country of provenance
2. Age
3. Children
4. Occupation
5. Years of education
6. Legal status
7. How long in Italy
8. General perception
9. Cultural differences-social fabric
10. General condition of vulnerability

*Professionals*

1. Background professionale
2. How long in the migrant world
3. Tasks/areas of activity

*NGOs*

1. NGOs/3d sector areas of activity (general)

Patient-specific factors influencing access to care

1. Health literacy/Being informed about italian health system
2. Health literacy/Navigating the NHS (*Knowledge of who to turn to*)
3. Health literacy/Previous experience influencing the choices
4. Health literacy/Awareness of their own rights
5. Health literacy/Digital literacy
6. Ability to perceive their health needs and awareness of own health status *(also mental)*
7. Coexisting pressing issues
8. Legal status
9. Fear or Trust in the system
10. Out-of-pocket payment
11. Social determinants of health (housing, occupation, gender)
12. Physical accessibility
13. Social networks
14. Language-related aspects (*being able to communicate effectively and being understood*)
15. Cultural aspects (*also difference with health system from country of origin, specialized care, religious beliefs)*
16. Presence of an NGO

Health system’s factors influencing access to care

1. Influence of past experiences in the NHS (*can be positive, can be negative)*
2. Appropriateness of information given (*giving info on navigation, rules, rights, laws, Internet, health needs*)
3. Appropriateness of services provided
4. Operator-related
5. Healthcare workers (*racism, discrimination and pre-existing cultural stereotypes of social workers, good predisposition, bias)*
6. Healthcare workers/Professional and friendly behaviour
7. Healthcare workers/Workload
8. Healthcare workers/Distancing
9. HCW/lack or presence of training and competencies
10. Presence of a cultural mediator
11. Role of a cultural mediator
12. Advantages of a cultural mediator
13. Availability of health facilities and services
14. Waiting lists
15. Lack of care for psychological aspect/mental health

Italian structural, social and political factors influencing access to care

1. Intersectoral coordination
2. Collaboration among NGOs/3rd sector
3. Presence of collaboration between public health system and 3d sector (*NGO*)
4. Necessity of organizations from the 3rd sector
5. Social disinformation, ignorance, racism
6. Legal aspects - international law and universality
7. Legal aspects - Health access being dependent on administrative aspects
8. Political will: lack or presence
9. Funding - Insufficient funding or support from public institutions impacting the quality of health services
10. Health system policy - Changes post COVID
11. Health system policy - Structural adequacy on the Italian territory
12. Health system policy- burocrazia, laws often changing, no stability
13. Paradoxical situations

Consequences of a lack of regular access

1. Emergency department
2. Resorting to natural remedies
3. Self-care
4. Forgoing care
5. Going private if you are privileged
6. Social consequences

The invisibles/The unreached

1. Lack of access to any service (when they are isolated, or they cannot be reached by prevention campaigns, or health and social services, Fear preventing them to access, Communities resorting to self-care → no access to public services nor to NGOs

Suggestions/Recommendations to improve access to care
