# Supplementary material for: Navigating barriers and building solutions: a mixed-methods study on sexual and reproductive healthcare for migrant women in Milan
Source: Prim Health Care Res Dev. 2026 Feb 27;27:e29. doi: 10.1017/S1463423626100954 (PMC12964159; doi:10.1017/S1463423626100954)
Supplement: Marro et al. supplementary material 5 — Marro et al. supplementary material [file S1463423626100954sup005.docx]

**Annex 5: Multivariable analysis**

We conducted a multivariable logistic regression to further explore the factors associated with accessing EMERGENCY services for SRH-related consultations among migrant women in Milan. The model specification, treatment of missing data, and full results are reported here for transparency and replicability. The binary outcome variable was access to SRH care (SRH-related consultation vs. all other consultations). Predictors were selected based on their potential relevance to SRH access: income (yes vs. no), age (in tertiles), nationality (top four countries of birth vs. all others), Italian language proficiency (sufficient vs insufficient), years of residence in Italy (>3 vs. ≤3 years), SSN entitlement (yes vs no) and marital status/living arrangement (co-residing with a partner vs. not). Missing values were substantial in some variables, reflecting the real-world clinical data collection setting. To address this, we applied two complementary strategies. First, we retained missing values as separate categories (*missing indicator method*) to avoid case deletion and preserve sample size. Second, as a sensitivity analysis, we performed multiple imputation by chained equations (MICE) using the R *mice* package (van Buuren and Groothuis-Oudshoorn, 2011), assuming data were missing-at-random. Five imputed datasets (m=5) were generated. The imputed models yielded results consistent with the missing indicator models, supporting the robustness of findings. In each dataset, we fitted the same multivariable logistic regression model and pooled the parameter estimates displayed in the Table A1.3.

**Table A1.1:** *Distribution of outcome and predictor variables in the multivariable regression model*

| **Variable** | **Number** | **Percentage** |
| --- | --- | --- |
| **SRH diagnosis** | 3713 |  |
| No | 3289 | 88.6% |
| Yes | 424 | 11.4% |
| **Income** | 3713 |  |
| Yes | 962 | 25.9% |
| No | 2294 | 61.8% |
| Missing data | 457 | 12.3% |
| **Age group** | 3713 |  |
| 0-33 | 1307 | 35.2% |
| 34-47 | 1233 | 33.2% |
| >47 | 1173 | 31.6% |
| **Country** | 3713 |  |
| Others/missing | 1316 | 35.4% |
| Romania | 702 | 18.9% |
| Morocco | 644 | 17.3% |
| Peru | 815 | 22% |
| Egypt | 236 | 6.3% |
| **Knowledge of Italian language** | 3713 |  |
| Sufficient | 1793 | 48.3% |
| Insufficient | 1688 | 45.5% |
| Missing data | 232 | 6.25% |
| **Marital status** | 3713 |  |
| Co-residing | 1287 | 34.7% |
| Non-Co-residing | 2196 | 59.1% |
| Missing data | 230 | 6.2% |
| **Permanence in Italy** | 3713 |  |
| < 3 years | 1610 | 43.4% |
| > 3 years | 1665 | 44.8% |
| Missing data | 438 | 11.8% |
| **Entitlement to SSN enrollment** | 3713 |  |
| Yes | 1410 | 38% |
| No | 1980 | 53.3% |
| Missing data | 323 | 8.7% |

On average, women reporting no income had higher odds of accessing SRH-related consultations compared with those reporting an income (OR = 1.41, 95% CI 1.03–1.92, p = 0.033), adjusting for the other variables in the model. Women not co-residing with a partner were less likely to access SRH care (OR = 0.53, 95% CI 0.42–0.68, p < 0.001). Nationality also played a role: women from Morocco (OR = 2.18, 95% CI 1.51–3.13, p < 0.001) and Romania (OR = 2.73, 95% CI 1.91–3.91, p < 0.001) had significantly higher odds of SRH-related consultations compared with women from other countries. The imputed models yielded results consistent with the missing indicator models, supporting the robustness of the findings. As a an exploratory analysis, the coefficients of the multivariable model should be interpreted with caution. Future work will use a causal framework to select the variables and interpret the coefficients as effect estimates (Westreich, 2013).

| **Table *A1.2:*** *Multivariable logistic regression (missing indicator method)* | | | | |
| --- | --- | --- | --- | --- |
| ***1.2– Access for SRH problems*** | | | | |
|  | **Univariate** | | **Multivariate** | |
| **Predictor** | **OR (95% CI)** | **P-value** | **OR** | **P-value** |
| **Income** |  |  |  |  |
| Yes (ref.) | - | - | - | - |
| No  Missing data | 2.25 (1.7– 2.98)  1.46 (0.97-2.18) | < 0.001  0.007 | 1.41 (1.03 – 1.92)  0.95 (0.58 – 1.55) | 0.033  0.839 |
| **Age** |  |  |  |  |
| 0-33 (ref.) | - | - | - | - |
| 34-47 | 0.26 (0.20 – 0.33) | <0.001 | 0.28 (0.22 – 0.36) | <0.001 |
| >47 | 0.01 (0.00 – 0.02) | <0.001 | 0.01 (0.00 – 0.002) | <0.001 |
| **Country**  Others/missing (ref.)  Romania  Morocco | -  4.51 (3.36– 6.04)  2.89 (2.11 – 3.97) | -  <0.001  < 0.001 | -  2.73 (1.91 – 3.91)  2.18 (1.51– 3.13) | -  <0.001  <0.001 |
| Peru | 1.25 (0.88 – 1.78) | 0.216 | 1.55 (1.03 – 2.33) | 0.035 |
| Egypt | 3.43 (2.28 – 5.16) | <0.001 | 1.52 (0.95 – 2.43) | 0.082 |
| **Knowledge of Italian language** |  |  |  |  |
| Sufficient (ref.) | - | - | - | - |
| Insufficient | 0.73 (0.59 – 0.90) | 0.004 | 0.87 (0.66 – 1.13) | 0.30 |
| Missing data | 0.42 (0.24 – 0.73) | 0.002 | 0.56 (0.07 – 4.2) | 0.57 |
| **Marital status** |  |  |  |  |
| Co-residing (ref.) | - | - | - | - |
| Non-Co-residing | 0.33 (0.27 – 0.41) | <0.001 | 0.53 (0.42 – 0.68) | <0.001 |
| Missing data | 0.29 (0.17 – 0.50) | <0.001 | 2.38 (0.38 –16.2) | 0.384 |
| **Permanence in Italy** |  |  |  |  |
| < 3 years (ref.) | - | - | - | - |
| > 3 years | 1.41 (1.14 – 1.75) | 0.001 | 1.13 (0.85– 1.50) | 0.404 |
| Missing data | 0.53 (0.34 – 0.81) | 0.004 | 0.60 (0.31 – 1.17) | 0.138 |
| **Entitlement to SSN enrollment** |  |  |  |  |
| Yes (ref.) | - | - | - | - |
| Not | 0.78 (0.63 – 0.97) | 0.024 | 0.91 (0.71 – 1.18) | 0.499 |
| Missing data | 0.85 (0.58– 1.24) | 0.389 | 0.95 (0.61–1.47) | 0.82 |

**Table A1.3:** *Multivariable logistic regression (MICE)*

| **Predictor** | **OR (95% CI)** | **p-value** |
| --- | --- | --- |
| **Income** |  |  |
| Yes (ref.) |  |  |
| Income: No | 1.6 (1.16 – 2.20) | 0.004 |
| **Age** |  |  |
| 0–33 (ref.) |  |  |
| 34–47 | 0.35 (0.27 – 0.45) | <0.001 |
| >47 | 0 (0 – 0.03) | <0.001 |
| **Knowledge of the Italian language** |  |  |
| Sufficient (ref.) |  |  |
| Insufficient | 0.81 (0.61 – 1.07) | 0.139 |
| **Permanence in Italy** |  |  |
| <3 (ref.) |  |  |
| >3 | 1.17 (0.87 – 1.57) | 0.301 |
| **Entitlement to SSN enrollment** |  |  |
| Entitled (ref.) |  |  |
| Not entitled | 0.92 (0.7 – 1.21) | 0.559 |
| **Marital status** |  |  |
| Co-residing (ref.) |  |  |
| Non co-residing | 0.54 (0.42 – 0.7) | <0.001 |
| **Country** |  |  |
| Others (ref.) |  |  |
| Romania | 2.57 (1.77 – 3.73) | <0.001 |
| Morocco | 2.15 (1.47 – 3.14) | <0.001 |
| Peru | 1.45 (0.95 – 2.22) | 0.086 |
| Egypt | 1.68 (1.03 – 2.75) | 0.039 |
